# Infection promotes Ser-214 phosphorylation important for generation of cytotoxic tau variants

**DOI:** 10.1096/fj.202300620RR

**Published:** 2023-07

**Authors:** Ron Balczon, Chung-Sik Choi, Althea deWeever, Chun Zhou, Meredith S. Gwin, Claire Kolb, C. Michael Francis, Mike T. Lin, Troy Stevens

**Affiliations:** 1 Department of Biochemistry and Molecular Biology, University of South Alabama College of Medicine, Mobile, Alabama, USA; 2 Center for Lung Biology, University of South Alabama College of Medicine, Mobile, Alabama, USA; 3 Department of Physiology and Cell Biology, University of South Alabama College of Medicine, Mobile, Alabama, USA

**Keywords:** cytotoxicity, endothelium, long-term potentiation, oligomeric tau, pneumonia, *Pseudomonas aeruginosa*

## Abstract

Patients who recover from hospital-acquired pneumonia exhibit a high incidence of end-organ dysfunction following hospital discharge, including cognitive deficits. We have previously demonstrated that pneumonia induces the production and release of cytotoxic oligomeric tau from pulmonary endothelial cells, and these tau oligomers can enter the circulation and may be a cause of long-term morbidities. Endothelial-derived oligomeric tau is hyperphosphorylated during infection. The purpose of these studies was to determine whether Ser-214 phosphorylation of tau is a necessary stimulus for generation of cytotoxic tau variants. The results of these studies demonstrate that Ser-214 phosphorylation is critical for the cytotoxic properties of infection-induced oligomeric tau. In the lung, Ser-214 phosphorylated tau contributes to disruption of the alveolar–capillary barrier, resulting in increased permeability. However, in the brain, both the Ser-214 phosphorylated tau and the mutant Ser-214-Ala tau, which cannot be phosphorylated, disrupted hippocampal long-term potentiation suggesting that inhibition of long-term potentiation was relatively insensitive to the phosphorylation status of Ser-214. Nonetheless, phosphorylation of tau is essential to its cytotoxicity since global dephosphorylation of the infection-induced cytotoxic tau variants rescued long-term potentiation. Collectively, these data demonstrate that multiple forms of oligomeric tau are generated during infectious pneumonia, with different forms of oligomeric tau being responsible for dysfunction of distinct end-organs during pneumonia.

## 1 ∣ INTRODUCTION

Nosocomial pneumonia is the leading cause of death from the hospital-acquired infections. Moreover, survivors of the initial infection exhibit elevated death rates following hospital discharge and various morbidities, including cognitive deficits.^1-3^ The reasons for these long-term consequences of pneumonia have not been defined, although our previous work has demonstrated that pulmonary infection induces the release of toxic amyloids from the pulmonary endothelium.^4,5^ These amyloids, which include oligomeric tau and Aβ, have been detected in the plasma, bronchoalveolar lavage fluid, and cerebrospinal fluid of animals and human patients following pulmonary infection,^6-10^ indicating that they exhibit hematogenous dissemination and access organs peripheral to the lung contributing to the cognitive deficits and potentially other morbidities associated with recovery from pneumonia.

Analysis of oligomeric tau variants released from endothelial cells following infection indicates that they are heat stable, cytotoxic, hyperphosphorylated, and transmissible between cells, animals, and species.^5-7,10^ In addition, they are long-lived and can be detected in the plasma at least 1 month following the initial infection.^10^ Application of infection-induced oligomeric tau to brain slices in vitro inhibits long-term potentiation (LTP; 6–8,10), an indicator of defective neuro-communication, and installation of oligomeric tau into the airways of animals leads to inhibition of neuro-communication as early as 24 h post-treatment with LTP defects persisting in animals for at least 1-month following treatment.^10^

Biochemical analyses have demonstrated that bacterial infection leads to phosphorylation of endothelial tau on Ser-214.^11,12^ The significance of this phosphorylation event has not been studied in detail, and whether phosphorylation of Ser-214 contributes to the pathogenicity of the infection-elicited endothelial tau is unknown.

During infection of endothelial cells by *Pseudomonas aeruginosa*, a common cause of hospital-acquired pneumonia, a group of bacterial exoenzymes is transferred from the bacterial cells to the cytoplasm of pulmonary endothelial cells via a type III secretion system.^13-15^ A critical exoenzyme, ExoY, is a nucleotidyl cyclase, which produces cyclic nucleotides within the cytoplasm of infected cells. Elevated levels of cyclic nucleotides lead to the activation of kinases within the infected cells.^16^ These kinases then can phosphorylate tau on Ser-214 and potentially other sites on the protein. Once hyperphosphorylated, tau can oligomerize and be released from cells.^4-10^

The studies outlined in this paper were designed to assess whether the phosphorylation at Ser-214 is important for production of cytotoxic oligomeric tau by endothelial cells. In support of this idea, immunodepletion of tau from infection-induced supernatants depleted cytotoxic activity. In addition, mutation of the Ser-214 to alanine significantly decreased the cytotoxic activity of supernatants produced following infection and the ability of supernatants to disrupt the pulmonary endothelial barrier. Inhibition of protein kinase A (PKA) activity decreased both Ser-214 phosphorylation levels and cytotoxic activity of secreted tau. In contrast to its effects on cytotoxicity and pulmonary barrier integrity, mutation of Ser-214 did not, although global dephosphorylation of tau did, inhibit the ability of infection-induced cell culture supernatants to disrupt LTP in a rodent brain slice model. Collectively, these data demonstrate a role for Ser-214 phosphorylation of tau, perhaps through activation of PKA, in the generation of cytotoxic forms of secreted tau. Moreover, the results indicate that multiple forms of phosphorylated oligomeric tau, some of which appear to have distinct effects, are generated by endothelium during infection.

## 2 ∣ METHODS

### Bacterial strains—

Two strains of *Pseudomonas aeruginosa* were used in these studies. The ExoY^+^ strain (PA103Δ*exoUexoT*::Tc/pUCP-*exoY*) expresses exclusively the ExoY exoenzyme. This strain has been described previously.^17^ The PA103 strain expresses exoenzymes ExoU and ExoT, and it was characterized previously.^18^

### Animals—

Experimentation with mice and rats was approved by the Institutional Animal Care and Use Committee of the University of South Alabama and conducted in accordance with the “Guide to the Care and Use of Laboratory Animals.”

### Cell culture—

Primary cultures of rat pulmonary microvascular endothelial cells (^19,20^:PMVECs) were used for production of cytotoxic supernatants and analysis of cytotoxicity. The cells were maintained in Dulbecco's Modified Eagle's Medium (DMEM) containing 10% fetal bovine serum and maintained at 37°C in 5% CO_2_ as detailed previously.^5,21,22^ In addition to wild-type PMVECs, various modified strains of PMVECs were also used. Specifically, PMVECs in which the tau gene was disrupted using Crispr/Cas9 (Tau KO cells) were used for some studies.^21^ In addition, Tau KO cells in which the cDNA encoding the 0N4R, 1N4R, 2N4R, or big tau isoform of rat PMVEC tau were also used, as well as a strain of tau KO cells in which the empty vector was used for transfection. Cloning of rat lung tau and generation of tau KO PMVECs were described previously.^21^

### Generation of stable cell lines—

To rescue tau, individual tau cDNAs were overexpressed separately in a *MAPT* KO cell clone and stable cell lines were created. Briefly, 1 day after seeding tau KO cells, DNA plasmid containing an individual tau endothelial tau cDNA construct (rat pulmonary endothelial cells express 0N4R, 1N4R, 2N4R, and big tau forms of tau)^21^ was transfected into cells using FuGENE HD (Promega #E2311) according to the manufacturer's recommendations. Once the cells reached ~95% confluency, the medium was replaced with complete medium containing 500 μg/mL of G418 (Geneticin; Fisher #BP6731). Cell medium was replaced every two to 3 days until stable colonies were established and selected. Selected clones were analyzed for tau expression by western blot.

### Site-directed mutagenesis—

To generate the mutant 1N4R tau harboring a serine 214 to alanine mutation (S214A), site-directed mutagenesis was performed using Quickchange II XL Site-Directed Mutagenesis Kit (Agilent Technologies #200523) according to the manufacturer's instructions. Primers used in the PCR were as follows: Forward-CCCGTACCCCA**GCC**CTACCAACGCCG and Reverse-CGGCGTTGGTAG**GGC**TGGGGTACGGG. The PCR product was digested with HindIII and XbaI and inserted into pcDNA3.1/V5-HisA (Invitrogen). The sequences of the mutant constructs were verified by DNA sequencing. Construction of stable cell lines expressing S214A mutant 1N4R tau was performed as described above.

### Generation of cytotoxic supernatants—

Production of cytotoxic supernatants was performed as detailed previously.^5,22^ Briefly, a confluent 150 cm plate of PMVECs was infected with either ExoY or PA103 strain of *P. aeruginosa* diluted into Hank's Balanced Salt Solution (HBSS) at an MOI of 20:1. The infection was allowed to proceed until visible gaps appeared in the cell monolayer (approximately 3.5 h for PA103 and 6 h for ExoY^+^ bacteria). At that time, the supernatant was collected, centrifuged at 3500 x g for 10 min to remove cellular debris, and then sterilized by passage through a 0.22 μm filter. The filter-sterilized supernatant was then either used immediately or stored at −80°C until use.

### Cytotoxicity assay—

To assess supernatant cytotoxicity, PMVECs were grown to confluence in 6-well dishes, the culture media were aspirated, and filter-sterilized supernatants were added to individual wells of the 6-well dish. The cells were incubated for 21–24 h and then photographed. Cell killing was quantified as detailed previously.^5,22^ The negative control for this experiment was to culture one of the six wells with HBSS alone. For some experiments, supernatants generated using WT PMVECs were immunodepleted using an antibody against Ser-214 (Abcam Cat# ab10891, RRID:AB_442836). For immunodepletion, 1 μL of antibody was added to 2.5 mL of cytotoxic supernatant, the preparation was incubated with mixing for 3 h, and then, 50 μL of protein A/G agarose was added (Pierce product #20421) for 3 h. The agarose beads were pelleted, and the supernatant was collected and placed at 95°C for 10 min to denature any residual antibody (cytotoxic oligomeric tau is heat stable, Balczon et al.^5^). The immunodepleted supernatant was filter-sterilized and then added to cells in a six-well dish as outlined above.

### Kinase assay—

To assess PKA activity, either uninfected PMVECs or PMVECs that were infected with either ExoY^+^ or PA103 bacteria as detailed above were treated as follows. When gaps appeared between infected cells in the monolayer, the control, and experimental cells were collected by trypsinization, pelleted, rinsed once with phosphate-buffered saline, and then resuspended in an extraction buffer^23^ composed of 20 mM Tris, pH 7.5, 150 mM NaCl, 1 mM EDTA, 1 mM EGTA, 1% Triton X-100, phosphatase inhibitors (Boston BioProducts product #BP-479), and a protease inhibitor cocktail of chymostatin, leupeptin, antipain, and pepstatin that were each at a final concentration of 1 μg/mL. Insoluble material was removed by pelleting at 14 000 × *g* for 20 min at 4°C, and the supernatant was retained. The concentration of protein in each supernatant was determined using Pierce BCA Protein Assay Kit components (Thermo Scientific product #23227), 250 μg of protein was collected from each supernatant and transferred to individual microfuge tubes, and the volume of each was brought to 1 mL using the extraction buffer. One microliter of anti-protein kinase A (PKA) antibody (Santa Cruz Biotechnology Cat# sc-28 315, RRID:AB_628136) was added to each tube, and the tubes were placed on a shaker at 4°C for 3 h. Fifty microliters of protein A/G agarose was added to each tube, and the tubes were returned to the shaker for 3 h. The agarose beads were then pelleted and rinsed six times with extraction buffer, once with extraction buffer containing 0.5 M NaCl, and then once with extraction buffer containing 0.1% Tween detergent. The agarose beads then were pelleted and rinsed once with kinase assay buffer (25 mM Tris, pH 7.5, 2 mM DTT, 10 mM MgCl_2_, and phosphatase inhibitors^22^), and the pelleted beads were then resuspended in 25 μL of kinases assay buffer. One microliter of human hippocampus brain homogenate (GeneTex product #GTX26445) was added to each tube, followed by ATP to a final concentration of 1 μM. The tubes were placed at 37°C for 5 min, and then, an equal volume of 2x SDS-PAGE sample buffer was added to each tube. The proteins were resolved by SDS-PAGE and transferred to nitrocellulose. The blots were then probed with anti-Ser 214 antibody (1:1000 dilution) followed by peroxidase-labeled anti-rabbit IgG (1:20 000; Abcam Cat# ab7090, RRID:AB_955417). Blots were developed using chemiluminescence (Thermo Scientific product #A38554).

#### PKA inhibition–

Inhibition of PKA activity was performed using previously reported methods.^24^ Briefly, the PKI-(6–22)-amide peptide (Santa Cruz Biotechnology #sc-201160) was delivered to PMVECs using the Chariot Delivery System (Active Motif North America product #30025) according to the manufacturer's instructions 2 h prior to infection of the cells with ExoY^+^ bacteria.

### PKA activation—

PKA was activated pharmacologically by treatment with forskolin and rolipram.^19,24^ Briefly, rolipram (10 μM) and forskolin (100 μM) were added to PMVECs, and the cells were cultured for up to 9 h. Control cells were treated with an equal volume of vehicle alone (100% EtOH). Both supernatants and cells were collected for analysis. Supernatants were dialyzed against HBSS to remove rolipram and forskolin and then were either added to cultured PMVECs to assess cytotoxicity or concentrated using a Centricon device and then analyzed by immunoblot. Cells were collected and lysed in kinase assay extraction buffer (see above), protein concentrations were determined, and then, the proteins were either analyzed by immunoblot or PKA was immunoprecipitated for performance of kinase activity assays as described above.

### Immunoblotting—

Immunoblot analysis was performed as detailed previously.^5,12,22^ Primary antibodies used included anti-Ser 214 (1:1000 dilution; Abcam product #ab10891), T22 anti-oligomeric tau (1:5000 dilution; EMD Millipore product #ABN454), and Tau-1 antibody (1:2000 dilution; Millipore-Sigma product #MAB3420; RRID:AB_94855), and secondary antibodies were peroxidase-labeled anti-rabbit IgG (1:20 000 dilution; Abcam product #ab7090; RRID:AB_955417) and antimouse IgG (1:10 000 dilution; Abcam product# ab97040, RRID:AB_10698223). The blots were developed using chemiluminescence procedures (Thermo Scientific product #A38554). Band intensities were quantified using ImageJ Fiji (RRID:SCR_002285).

### Analysis of $K_{f}$ in whole rat lungs treated with cytotoxic supernatant—

All experimental procedures were performed in accordance with current provisions of the US Animal Welfare Act and were approved by the Institutional Animal Care and Use Committee of the University of South Alabama. Permeability was assessed by filtration coefficient ($K_{f}$) in standard isolated lung experiments essentially as described previously.^5,25,26^ For these experiments, supernatant collected from either ExoY^+^-infected wild-type PMVECs, tau KO PMVECs, or tau KO PMVECs overexpressing either 1N4R tau or 1N4R-Ser214Ala tau was used. For each experiment, 10 mL of supernatant was concentrated to 150 μL using a Centricon microfiltration device with a 3 kDa cutoff (Merck Millipore product #UFC800324, Cork, Ireland). Female CD rats ranging from 10 to 15 weeks were purchased from Charles River Laboratories (Raleigh, NC). Animals were anesthetized using Fatal Plus (65 mg/kg body weight). Once a surgical plane was achieved, as defined by the absence of a withdrawal reflex following toe and tail pinch, animals were intubated and ventilated, a sternotomy was performed, and pulmonary artery and left atrium catheters were placed. Blood was taken by heart puncture from the right ventricle. Heart and lungs were removed *en bloc* and suspended in a humidified chamber where mechanical ventilation and blood flow were established. Rat lungs were perfused at constant flow (40 mL/min/Kg body weight) with buffer (in mmol/L: 119.0 NaCl, 4.7 KCl, 1.17 MgSO_4_, 1.0 Na_2_HPO_4_, 1.18 KH_2_PO_4_, 23 NaHCO_3_, and 5.5 glucose) containing 4% BSA/6% blood in 50 mL total volume and physiological (2.2 mmol/L) CaCl_2_ at pH 7.4 at 38°C. After the lungs were perfused for 15 min to reach an equilibrated status, a baseline $K_{f}$ was measured as previously described.^5^ $K_{f}$, the product of specific endothelial permeability and surface area for exchange, is a sensitive measure of lung endothelial permeability when surface area is fully recruited. $K_{f}$ was calculated as the rate of lung weight gain obtained 13 to 15 min after a 10 cm H_2_O increase in pulmonary venous pressure and normalized per 100 g predicted wet lung weight. After $K_{f}$ measurement, venous pressure was set back to the previous level, and 150 μL of a concentrated supernatant was slowly infused into the lung circulation by injection into the inflowing perfusate and circulated for a total of 4 h, during which period $K_{f}$ was measured again as described above at 2- and 4-h time points after supernatant injection. Data are reported as means ± SEM. Multiple *t*-test analysis was used to evaluate the differences between two groups. Significance was considered at *p* ≤ .05.

### Hippocampal slice preparation—

Hippocampal slices were prepared from adult 9–13-week-old wild-type (C57BL/6J; RRID:IMSR_JAX:000664) mice. Upon obtaining the brains from the animals after euthanasia, the brains were submerged in an ice-cold sucrose-artificial cerebrospinal fluid (aCSF) solution (in mM): 70 glucose, 80 NaCl, 2.5 KCl, 21.4 NaHCO_3_, 1.25 NaH_2_PO_3_, 0.5 CaCl_2_, 7 MgCl_2_, 1.3 ascorbic acid, and 20 glucose. After 1 min of incubation, the hippocampi, obtained by removing the cerebral cortices, were placed on an agar block, transferred into a slicing chamber (Leica VT1200s, Leica Instruments), and the prepared transverse slices (300 μm) were placed in a holding chamber containing regular aCSF (in mM): 125 NaCl, 2.5 KCl, 21.5 NaHCO_3_, 1.25 NaH_2_PO_4_, 2.0 CaCl_2_, 1.0 MgCl_2_, and 15 glucose. Slices were incubated at 35°C for 30 min and then at room temperature for >1 h before field potential recordings were performed at room temperature. All solutions were constantly equilibrated with carbogen (95% O_2_ and 5% CO_2_).

### Electrophysiological recordings—

Prior to recording, hippocampal slices were incubated in endothelial supernatants equilibrated in carbogen for 90 min. After the incubation, slices were transferred to a recording chamber with continuous superfusion (1 mL/min) of aCSF containing SR95531 (2 μM; Sigma-Aldrich #505986) and CGP55845 (1 μM; Sigma-Aldrich #SML0594) to block inhibitory synaptic transmission. Hippocampal slices were visualized using a fixed-stage upright microscope (Leica) equipped with infrared differential interference contrast optics. Both recording and stimulating electrodes were pulled from borosilicate glass pipettes (BF150-86-10, Sutter Instruments) with tip resistances of 1 and 0.6 MΩ, respectively. With the CA3 region cut away to eliminate recurrent excitation within the CA3 subfield, the stimulating electrode, connected to an ISO-Flex stimulation isolation unit (A.M.P.I.), was placed in the CA1 stratum radiatum (~100 μm from the somata) to stimulate the CA3 axon collaterals. The stimulus duration was 0.1 ms, allowing for clear separation of fiber volley from the preceding stimulus artifact.

Recordings were obtained using an EPIC10 amplifier (HEKA). Analog signals were further amplified 10 X and filtered at 5 kHz using an Axopatch amplifier (Axon Instruments) and digitized at 20 kHz using Patchmaster software (HEKA; RRID:SCR_000034). Evoked field excitatory postsynaptic potentials were recorded every 20 s. Following a stable field excitatory postsynaptic potential of >10 min, a theta-burst stimulation, consisting of three sweeps (a single burst consists of five stimuli delivered at 100 Hz and 10 bursts delivered at 5 Hz per sweep) delivered at 30 s intervals, was delivered to induce synaptic strengthening. Long-term potentiation (LTP) was calculated from 55 to 60 min after the theta-burst stimulation and normalized to the baseline field excitatory postsynaptic potential.

### Phosphatase treatment—

Dephosphorylation of proteins was conducted using lambda protein phosphatase (Lambda PP, New England BioLabs #P0753S) following the manufacturer's instruction. In brief, 1N4R-overexpressing endothelial cells were infected for 6 h with ExoY^+^ (MOI of 10:1), washed twice with cold PBS, lysed in the recommended lysis buffer (20 mM Tris–HCl, 150 mM NaCl, 1 mM EDTA, 1 mM EGTA, 1% Triton X-100, pH 7.5) including Halt protease inhibitor cocktail (Thermo Scientific), collected in the lysis buffer, rotated at 4°C for 30 min, sonicated using a Virsonic 60 (VirTis) at 10 watts for 15 s, and centrifuged at 14 000 rpm for 15 min. The supernatants were collected for analyses. Lysates were treated with or without 400 units of Lambda PP at 30°C for 30 min. Samples were then heated at 65°C for 60 min to inactivate the enzymes. Dephosphorylated tau proteins were confirmed by western blotting using total tau and phospho-specific tau antibodies. Control and phosphatase-treated supernatants were then added to brain slices and LTP measurements were performed as outlined above.

### Bimolecular fluorescence complementation (BiFC) measurement—

BiFC studies were carried out as we previously described.^21^ Briefly, HEK293 cells stably expressing human neural 2N4R BiFC probes were used. Cells grown at 70% confluence in 12-well plates containing coverslips were incubated with 8-pCPT-cAMP (15 μM), 8-pCPT-cGMP (15 μM), vehicle (Opti-MEM), or were transfected with tau seeds prepared from control or ExoY^+^-infected PMVEC (i.e., WT, tau KO, 1N4R-overexpressing, and S214A-overexpressing PMVECs) supernatants using the Xfect TM Protein Transfection Reagent (Clontech Lab #NC1102016). Coverslips were mounted after cell fixation in 4% paraformaldehyde solution for 15 min at room temperature, 24 h after transfection. Acquisition of the fluorescence signal was performed using a Zeiss LSM 980 Airyscan confocal microscopy and a 10X objective. The BiFC signal was acquired using excitation wavelength at 514 nm and emission wavelength at 535 nm and analyzed using FIJI-ImageJ and quantified as we previously described.^21^ Three images were captured for each sample, and the fluorescence signals from these images were averaged. Tau-BiFC signal was expressed as a percentage by normalizing the fraction of cell area with high fluorescence intensity to the fraction of cell area from vehicle controls performed in parallel.

### Data and statistical information—

For all studies, means were compared using Student's *t*-test or ANOVA followed by Tukey's post hoc analyses. Differences were considered significant at *p* ≤ .05. For electrophysiologic data, offline analyses were performed using custom macros written in Igor Pro (WaveMatrics; IGOR Pro, RRID:SCR_000325). Field excitatory postsynaptic potential amplitude was determined from the slope measured between 10 and 50% of the rising phase. Data were binned at 3-min intervals to generate summary field excitatory postsynaptic potential slope plots and are expressed as mean ± SEM.

## 3 ∣ RESULTS

**FIGURE 1:**
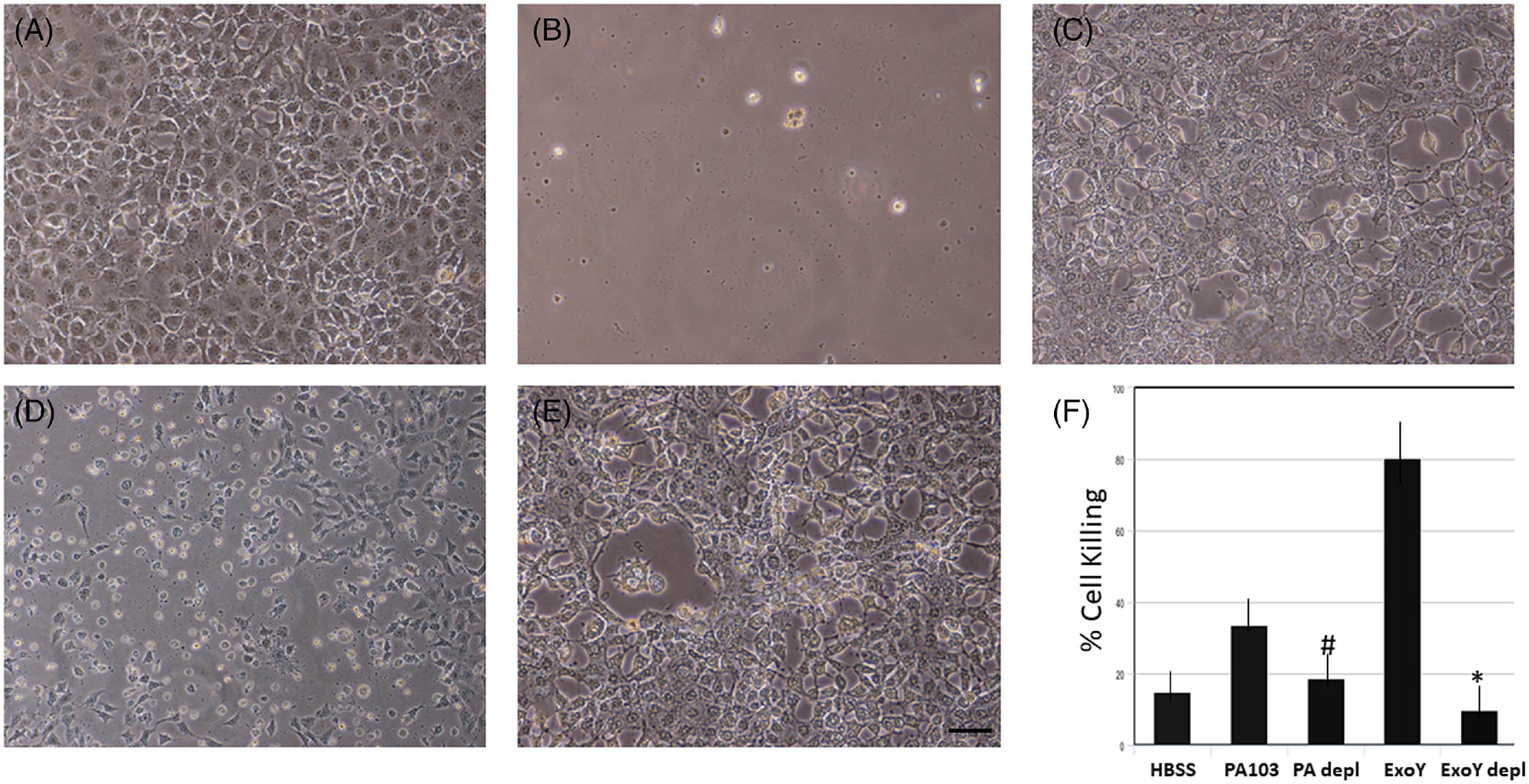
Immunodepletion of Ser-214 tau from supernatants generated following *P. aeruginosa* infection of rat PMVEC depletes cytotoxic activity of the supernatants. Cytotoxicity was assessed after incubation of naïve PMVECs treated with supernatant collected from PMVECs that had been infected with either ExoY^+^ (B and C) or PA103 strains of *P. aeruginosa* (D and E). Both untreated supernatant (B and D) and supernatant that was immunodepleted using anti-Ser-214 antibody (C and E) were used. Cells incubated with HBSS alone (A) were used as a negative control. Quantitation of cell killing is shown (F). * denotes *p* ≤ .05 when comparing B to C; # denotes *p* ≤ .05 when comparing D to E; *N* = 4. Bar = 50 μm.

**FIGURE 2:**
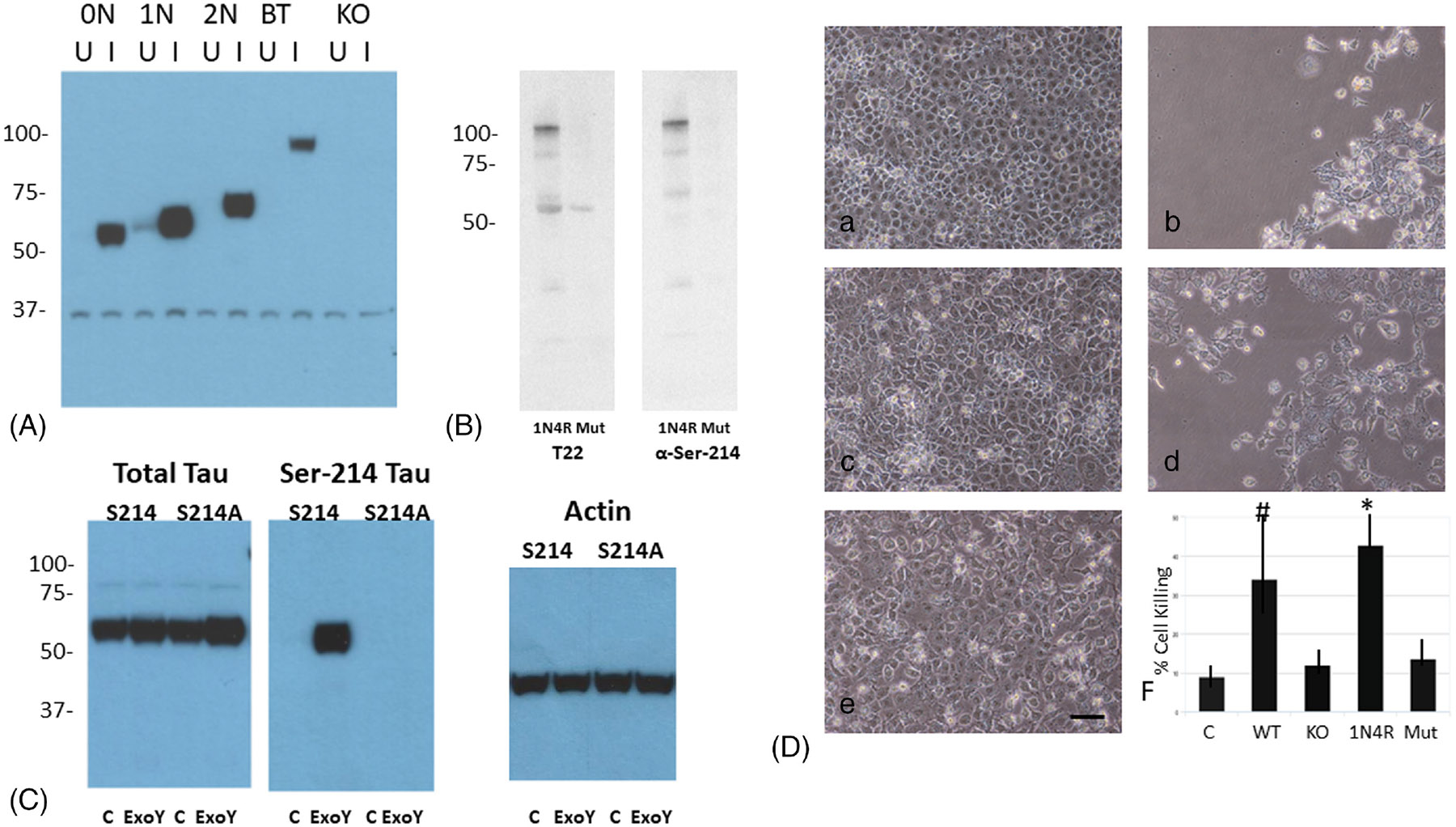
Mutation of Ser-214 to Ala decreases the production of cytotoxic oligomeric tau following *P. aeruginosa* infection. (A) Immunoblot analysis for Ser-214 tau in whole-cell extracts prepared from tau knockout cells (KO) and KO cells that were transfected individually with constructs encoding either ON4R, 1N4R, 2N4R, or big tau (BT). Cell extracts were prepared from both uninfected cells (U) and cells that had been infected for 4 h with ExoY^+^ bacteria (I). M_r_ in kDa. (B) Immunoblot analysis of supernatant collected from PMVECs expressing either wild-type 1N4R tau (1 N4) or mutated Ser-214 tau (Mut) PMVECs following infection by ExoY^+^ bacteria using T22 anti-oligomeric tau antibody and anti-Ser-214 antibody. M_r_ in kDa. (C) Immunoblot analysis of nonmutated 1N4R-expressing PMVECs (S214) and mutant 1N4R expressing PMVECs (S214A) for total tau expression using Tau1 antibody (Left panel). Both uninfected (C) and ExoY^+^-infected cells (ExoY) were analyzed. The same samples were also analyzed by immunoblot using antibody specific for Ser-214 tau. Tau in mutant cells could not be phosphorylated on Ser-214 (middle panel). The same samples were analyzed by immunoblot using Actin antibody as a loading control (right panel). M_r_s in kDa. (D) Analysis of cytotoxicity of PMVECs expressing 1N4R tau and 1N4R tau mutated at Ser-214. Supernatant was collected from WT PMVECs (b), tau KO PMVECs (c), tau KO PMVECs expressing 1N4R tau (d), and tau KO PMVECs expressing 1N4R tau mutated at Ser-214 (e) following infection by *P. aeruginosa* strain ExoY^+^. The supernatants were applied to naïve PMVECs and cell killing was quantified (f). Cells treated with HBSS served as a negative control (a). * denotes *p* ≤ .05 when comparing 1N4R to Mut and # denotes *p* ≤ .05 when comparing wild type to KO.

Toxicity assays using supernatants generated following infection of PMVECs by *P. aeruginosa* were performed to determine whether Ser-214 phosphorylation of tau is required for the cytotoxic activity of oligomeric tau. As shown in Figure 1, immunodepletion of Ser-214 tau from cytotoxic extracts produced following infection of PMVECs with either ExoY^+^ or PA103 strains of *P. aeruginosa* greatly reduced the killing activity of the extracts. To investigate the importance of Ser-214 phosphorylation for the generation of cytotoxic tau more rigorously, site-directed mutation of the Ser-214 phosphorylation site on tau was performed, and a PMVEC cell-line expressing exclusively mutant Ser-214 tau was generated by the first knocking out the endogenous tau gene in PMVECs and then generating stable transfectants that expressed individual tau isoforms. Once produced, each cell line was infected with ExoY^+^ bacteria, and then, Ser-214 phosphorylation levels were assessed by immunoblot analysis. Previously, we had demonstrated that there is a basal level of Ser-214 phospho-tau in endothelial cells, and the level of phospho-tau increased following infection by ExoY^+^ bacteria.^11^ As shown in Figure 2A, the 1N4R isoform of tau was the only form exhibiting this physiological behavior. Whereas all four isoforms of tau exhibited phosphorylation on Ser-214 following infection by ExoY^+^ bacteria, only the 1N4R form of endothelial tau exhibited baseline phosphorylation in the uninfected state. The other three tau isoforms, 0N4R, 2N4R, and big tau, did not exhibit tau phosphorylation in uninfected cells. As expected, tau knockout cells did not exhibit phospho-tau in either the uninfected or infected states (Figure 2A). As such, endothelial cells expressing the 1N4R form of tau were used for analyses of Ser-214 function.

To analyze the function of hyperphosphorylated Ser-214 forms of tau, Ser-214 was converted to a nonphosphorlatable Ala residue, a plasmid containing the mutant form of 1N4R tau was transfected into tau KO cells, and stable transfectants were selected. Once generated, cells expressing the Ser-214 mutant form of 1N4R tau were infected with ExoY^+^ bacteria and supernatant was collected. To assess whether oligomeric forms of tau are generated by Ser-214 mutant cells, immunoblot analysis was performed using an antibody that recognizes all forms of oligomeric tau (T22). As shown in Figure 2B, supernatant generated following ExoY^+^ infection of Ser-214 mutant cells produced supernatant with greatly reduced amounts of oligomeric tau when compared with the amounts secreted from cells expressing nonmutated 1N4R tau. As expected, supernatant produced by the Ser-214 mutant cells did not secrete oligomeric tau containing Ser-214, whereas cells expressing nonmutated 1N4R tau secreted multiple different forms of Ser-214-containing oligomeric tau. To verify that the decreased levels of T22-reactive oligomeric tau were not due to decreased expression of S214A tau relative to the levels of tau produced by 1N4R expressing cells, cells were analyzed for tau expression using a pan-tau antibody (Tau 1), and similar levels of tau expression were detected in both uninfected and ExoY^+^-infected PMVECs (Figure 2C). As expected, Ser214A tau could not be phosphorylated on Ser214 following infection, whereas nonmutated 1N4R tau could be phosphorylated (Figure 2C). As shown in Figure 2D, cytotoxic activity of supernatants generated following infection of Ser-214 mutant cells was greatly reduced when compared to the activity of supernatants generated following infection of either wild-type PMVECs or PMVECs expressing the 1N4R form of tau (Figure 2D). Supernatants collected from tau knockout cells transfected with a control empty vector construct exhibited little cytotoxic activity (Figure 2D), with remaining cytotoxicity most likely due to β-amyloid which we have previously shown to be released following infection.^5-10^

**FIGURE 3:**
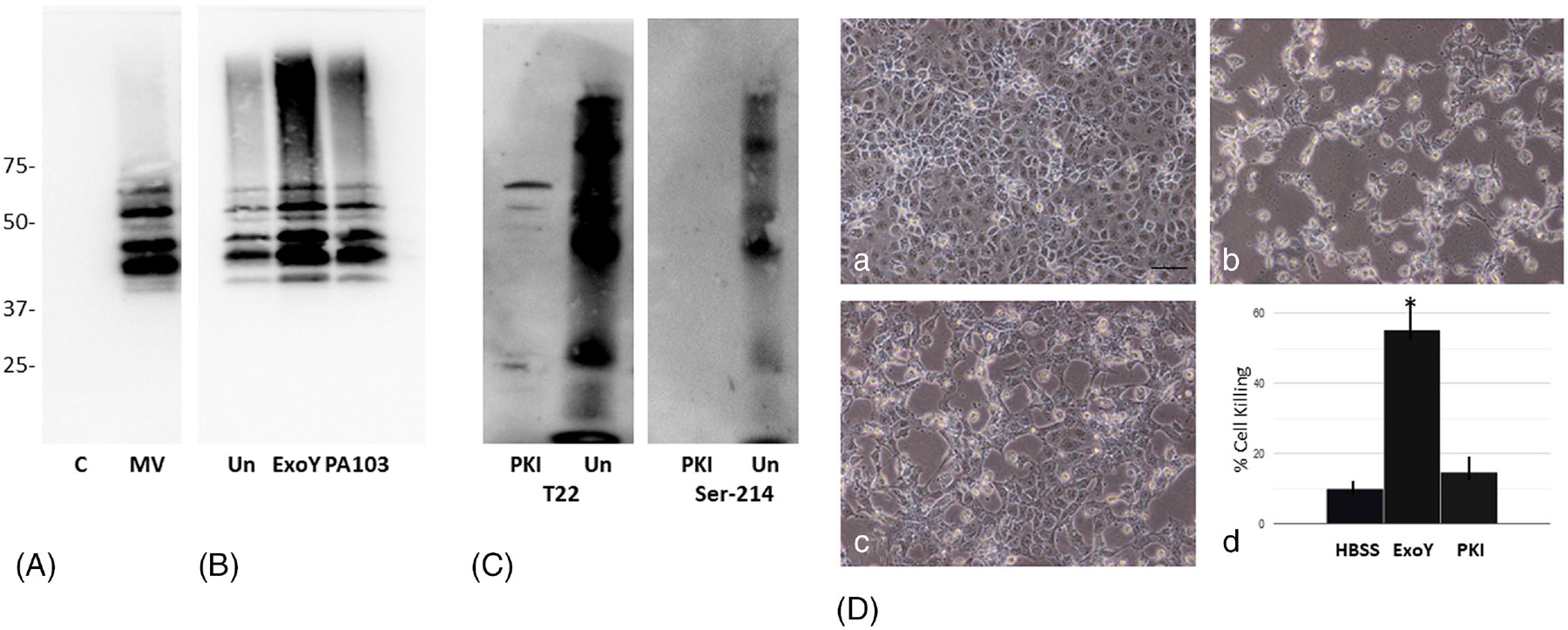
*Pseudomonas aeruginosa* infection leads to activation of PKA contributing to production of cytotoxic tau oligomers. (A) Immunoblot analysis of human hippocampal extract using antibody against Ser-214 tau failed to identify Ser-214 phosphorylated tau in the untreated control brain homogenate (C). PKA was immunoprecipitated from PMVEC extract and then incubated with ATP and hippocampal homogenate, and then, the mixture was analyzed by immunoblot analysis using antibody against Ser-214 tau (MV). Incubation of the brain extract with PKA immune-isolated from uninfected PMVECs demonstrated that PMVEC PKA can phosphorylate Ser-214 of tau. (B) PKA isolated from PMVECs that had been infected with either ExoY^+^ or PA103 strains of *P. aeruginosa* was more effective at phosphorylating brain tau than PKA isolated from uninfected (Un) control PMVECs. PKA was immunoprecipitated from untreated control PMVECs (Un) and PMVECs that were infected with either ExoY^+^ (ExoY) or PA103 (PA103), the immunoprecipitated PKAs were incubated with hippocampal extract and ATP, and Ser-214 phosphorylation was measured by immunoblotting using antibody against Ser-214 tau. Ser-214 phosphorylation by PKA isolated from ExoY^+^- and PA103-infected cells was increased 2.00 ± 0.79-fold and 1.98 ± 0.37-fold, respectively, relative to PKA-isolated untreated cells. Both values represent a significant increase in kinase activity (*p* ≤ .05, *N* = 7, ANOVA). (C) Inhibition of PKA using the PKI inhibitor blocked the release of hyperphosphorylated tau oligomers. Untreated control PMVECs (Un) and PMVECs treated with the PKI inhibitor (PKI) were infected with ExoY^+^. Supernatants were collected and then analyzed by immunoblot using T22 anti-oligomeric tau antibody and anti-Ser-214 tau antibody. M_r_s in kDa. (D) Inhibition of PKA decreased cytotoxic activity of supernatants generated by ExoY^+^ infection of PMVECs. PMVECs were treated with either HBSS (a), control ExoY^+^ supernatant (b), or supernatant that was collected from PMVECs that were pretreated with PKI prior to infection with ExoY^+^ (c). Quantitation of cell killing is shown (d). * denotes *p* ≤ .05 when comparing supernatant collected from untreated ExoY^+^ infected cells to supernatant from cells pretreated with PKI prior to infection, *N* = 3.

Studies then were performed to assess whether *P. aeruginosa* infection of rat PMVECs leads to activation of PKA. For this, an in vitro kinase assay was developed which used brain tau as the kinase target. As shown in Figure 3A, the commercially available human brain preparation exhibited no detectable Ser-214 phosphorylation of tau, and low levels of PKA activity capable of phosphorylating tau were detected in untreated PMVECs. However, infection of PMVECs with the strains ExoY^+^ and PA103 led to robust activation of PKA (Figure 3B), with infection by either strain leading to increased Ser-214 phosphorylation of tau relative to that detected using PKA purified from uninfected control PMVECs (2.00 ± 0.79-fold increase for ExoY^+^-infected cells, *p* ≤ .05; 1.98 ± 0.37-fold increase for PA103-infected cells, *p* ≤ .05, *N* = 7). To assess whether PKA activation is required for Ser-214 phosphorylation and the generation of cytotoxic oligomeric tau, PKA activity was inhibited using the PKI inhibitory peptide, and then, cells were infected with ExoY^+^. Immunoblot analyses of supernatant collected from PKI-treated cells demonstrated that levels of T22-reactive tau oligomers were greatly reduced, and Ser-214-containing oligomers could not be detected (Figure 3C). Cytotoxicity of supernatant generated from PKI-treated cells was assessed, and as shown in Figure 3D, inhibition of PKA activity greatly decreased the production and release of cytotoxic oligomeric tau. Collectively, these data support a mechanism whereby infection of PMVECs by *P. aeruginosa* contributes to increased PKA activity, Ser-214 phosphorylation of tau, and production and release of oligomeric, cytotoxic tau.

**FIGURE 4:**
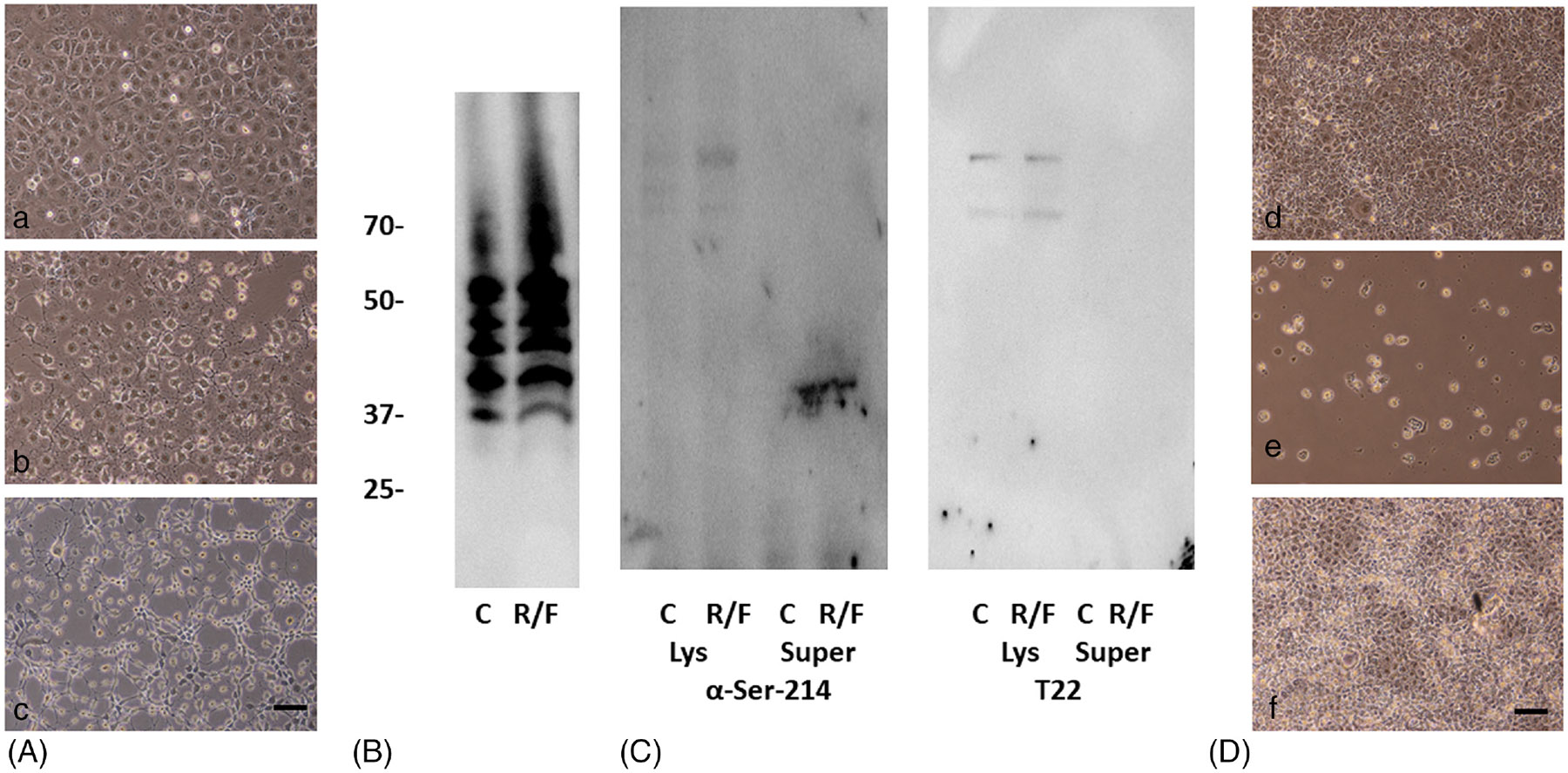
Pharmacological activation of PKA does not lead to generation of cytotoxic hyperphosphorylated tau. (A) Cultured PMVECs were either treated with rolipram (10 μM) and forskolin (100 μM) for 9 h (b) or incubated with ExoY^+^ bacteria for 6 h (c). Both treatments induced cell retraction and gap formation in the monolayer. An untreated control confluent monolayer of rat PMVECs is also shown (a). Bar = 50 μm. (B) Treatment with rolipram and forskolin leads to increased PKA activity. PKA was immunopurified from lysates generated from untreated control cells (C) and cells treated with rolipram and forskolin (R/F), and then, the immunopurified material was incubated with human brain extract along with ATP. Ser-214 tau levels then were measured by immunoblot using Ser-214 antibody followed by band intensity quantitation using ImageJ software, (*n* = 6, 1.39 ± 0.16-fold increase in PKA activity in the drug-treated cells, *p* ≤ .05, ANOVA). (C) Phospho-tau was not secreted from cells that were activated with rolipram and forskolin. Whole-cell lysates (Lys) and cell culture supernatants (Super) were collected from untreated control cells (C) and cells that were treated with rolipram and forskolin (R/F), and then, each sample was analyzed by immunoblot using either anti-Ser-214 antibody or T22 oligomeric tau antibody. Molecular weights in B and C are in kDa. (D) Cytotoxicity was assessed by adding supernatants collected from either ExoY^+^-infected PMVECs (b) or PMVECs that were treated with rolipram and forskolin (c). Control cells were incubated with HBSS alone (a). Cytotoxic activity was not detected in the supernatant from rolipram and forskolin-treated cells. Bar = 50 μm.

Experiments then were performed to assess whether stimulation of transmembrane adenylyl cyclases and inhibition of phosphodiesterases promotes activation of PKA sufficient to induce the production and release of cytotoxic tau from endothelial cells. For these studies, cells were treated with the pharmacological agents forskolin and rolipram, and then, the treated cells and cell supernatants were analyzed. Unlike short-duration treatment with rolipram and forskolin,^19,24^ the treatment of cells for extended time periods caused gaps to form in the cell monolayer that appeared similar in appearance to the gaps that form in monolayers following infection with ExoY^+^ bacteria (Figure 4A). PKA was isolated from control and experimental cells and then kinase assays were performed. As shown in Figure 4B, pharmacological treatment led to activation of PKA, with increased tau phosphorylation detected using PKA isolated from drug-treated cells when compared to PKA obtained from control cells (1.39 ± 0.16-fold PKA activation in treated cells relative to PKA from control cells, *N* = 4, *p* ≤ .05, ANOVA). Immunoblot analysis demonstrated a slight increase in Ser-214 phosphorylation of tau inside cells, although Ser-214 containing tau could not be detected in cell supernatants (Figure 4C). Likewise, T22 reactive material also was not detected in cell supernatants (Figure 4C). Finally, when supernatants from rolipram- and forskolin-treated cells were added to cultures of PMVECs, cytotoxic activity could not be detected. Collectively, these results indicate that even the maximal stimulation of transmembrane adenylyl cyclases and inhibition of phosphodiesterases,^19,24^ which increases cAMP and activates PKA is not sufficient for PMVECs to generate cytotoxic oligomeric tau, illustrating the privileged intracellular location of ExoY activity.

**FIGURE 5:**
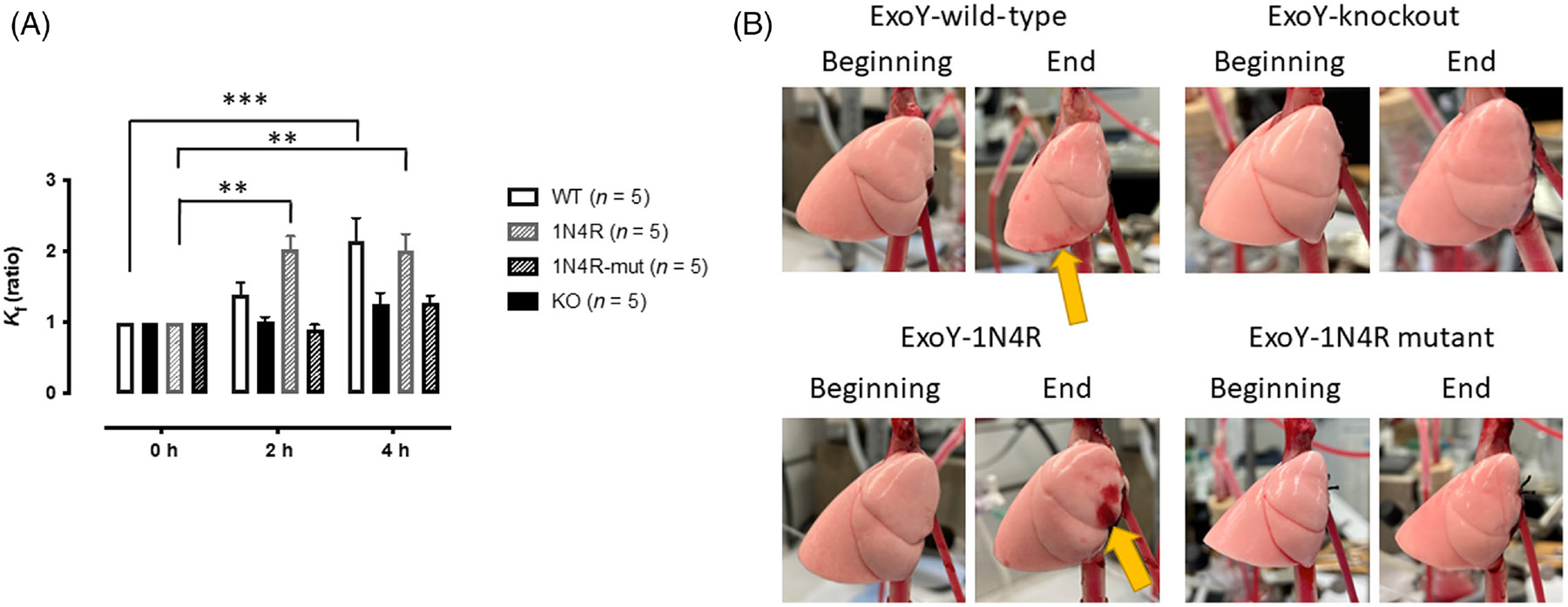
ExoY^+^ promotes Ser-214 phosphorylation of tau which is important for its lung cytotoxicity. (A) Supernatant was collected from wild type, tau knockout, 1N4R expressing, and Ser-214 mutant tau-expressing cells following ExoY^+^ infection, concentrated to 150 μL and then introduced into the circulation of isolated perfused rat lungs. Filtration coefficient ($K_{f}$) was recorded at 2 and 4 h postaddition of cytotoxic supernatant. Baseline $K_{f}$ among groups was 0.15 ± 0.02 g min^−1^ cm H_2_O^−1^ 100 g^−1^. * denotes *p* ≤ .05, using two-way ANOVA with Tukey's multiple comparison test. (B) Representative images of lungs at *T* = 0 (Beginning) and 4 h after the addition (End) of supernatants collected from ExoY^+^-infected wild type, tau knockout, 1N4R, and 1N4R mutant tau-expressing PMVECs. The yellow arrows show lung damage caused by the phospho-tau-containing supernatant.

Subsequent experiments were performed to examine whether Ser-214 phosphorylation of tau is required for other previously reported effects of endothelial-derived hyperphosphorylated tau. Specifically, studies were performed to assess whether phosphorylation of Ser-214 on endothelial tau is required for disruption of the pulmonary endothelial barrier^5^ and inhibition of long-term potentiation (LTP) using rodent brain slices^6,10^ as we had reported previously for hyperphosphorylated oligomeric tau. Supernatant generated following infection of wild-type PMVECs, tau knockout, 1N4R-expressing, and Ser-214 mutant 1N4R-expressing PMVECs were used to analyze each process. For pulmonary barrier studies, filtration coefficient ($K_{f}$) was measured using isolated perfused lungs ex vivo. Each supernatant was added individually to buffer that was transported through the circulation of an isolated lung, and then, the $K_{f}$ was measured at 2 and 4 h. As shown in Figure 5, supernatant collected from both infected wild-type and 1N4R-expressing PMVECs led to an increase in $K_{f}$, indicating disruption of the alveolar–capillary membrane and increased permeability. In contrast, supernatant collected from both KO cells and cells expressing Ser-214-Ala mutant 1N4R had little effect on $K_{f}$ (Figure 5) indicating limited disruption of the alveolar-capillary barrier.

**FIGURE 6:**
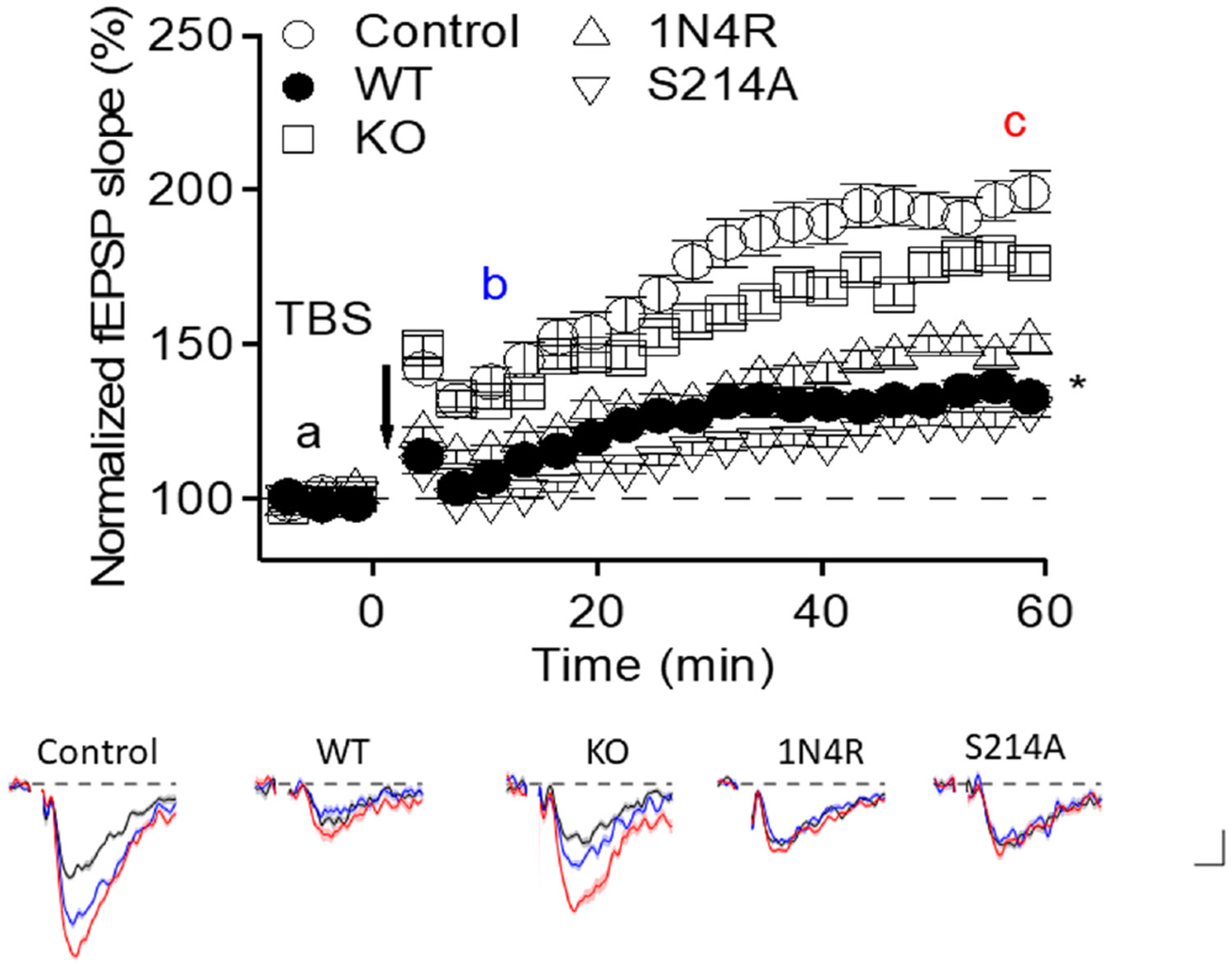
Endothelial cells release neurotoxic tau that inhibits synaptic plasticity. Summary plot of LTP. Field excitatory postsynaptic potential (fEPSP) slopes were normalized to those before the theta-burst stimulation (TBS; delivered at time 0) and plotted against time (mean ± SEM). Brain slices were untreated control (control) or treated with supernatants collected from wild-type (WT), tau knockout (KO), 1N4R overexpressing (1N4R), or Ser-214 to Ala mutant overexpressing (S214A) endothelial cells exposed to ExoY^+^. The responses from the control and KO treatment were significantly higher than the other groups (*F*(4,52) = 14.29; * indicates *p* ≤ .0001; ANOVA). The bottom shows representative averages of five traces obtained from timepoints “a” (pre-TBS; black), “b” (5–8 min post-TBS; blue), and “c” (57–60 min post-TBS; red). X-axis scale bar represents 2 ms and Y-axis scale bar represents 0.1 mV.

Effects on LTP were measured using an in vitro system. Each endothelial cell clone was infected with ExoY^+^ and supernatants were collected. Acute hippocampal slices prepared from mouse brains were incubated in individual endothelial supernatants for 90 min, and field potentials were recorded in the CA1 area, as we have previously described.^6,10^ After obtaining a stable baseline for 10 min, a theta-burst stimulation protocol was used to evoke LTP, and the amount of potentiation was determined 1 h later (Figure 6). Our results showed that while supernatant collected from infected wild-type PMVECs abolished LTP, the supernatant collected from tau KO PMVECs had minimal effect. As expected, supernatant collected from endothelial cells overexpressing 1N4R abolished LTP. Interestingly, the supernatant obtained from Ser-214-Ala mutant cells also inhibited LTP (Figure 6), indicating that synaptic plasticity is sensitive to oligomeric forms of the 1N4R tau isoform released from endothelial cells after ExoY^+^ infection, but insensitive to Ser-214 phosphorylation of tau (*F*(4,52) = 14.29, *p* ≤ .05, ANOVA; Tukey's post hoc test: control versus wild-type, *p* ≤ .05; control versus 1N4R: *p* ≤ .05; control versus Ser-214, *p* ≤ .05).

**FIGURE 7:**
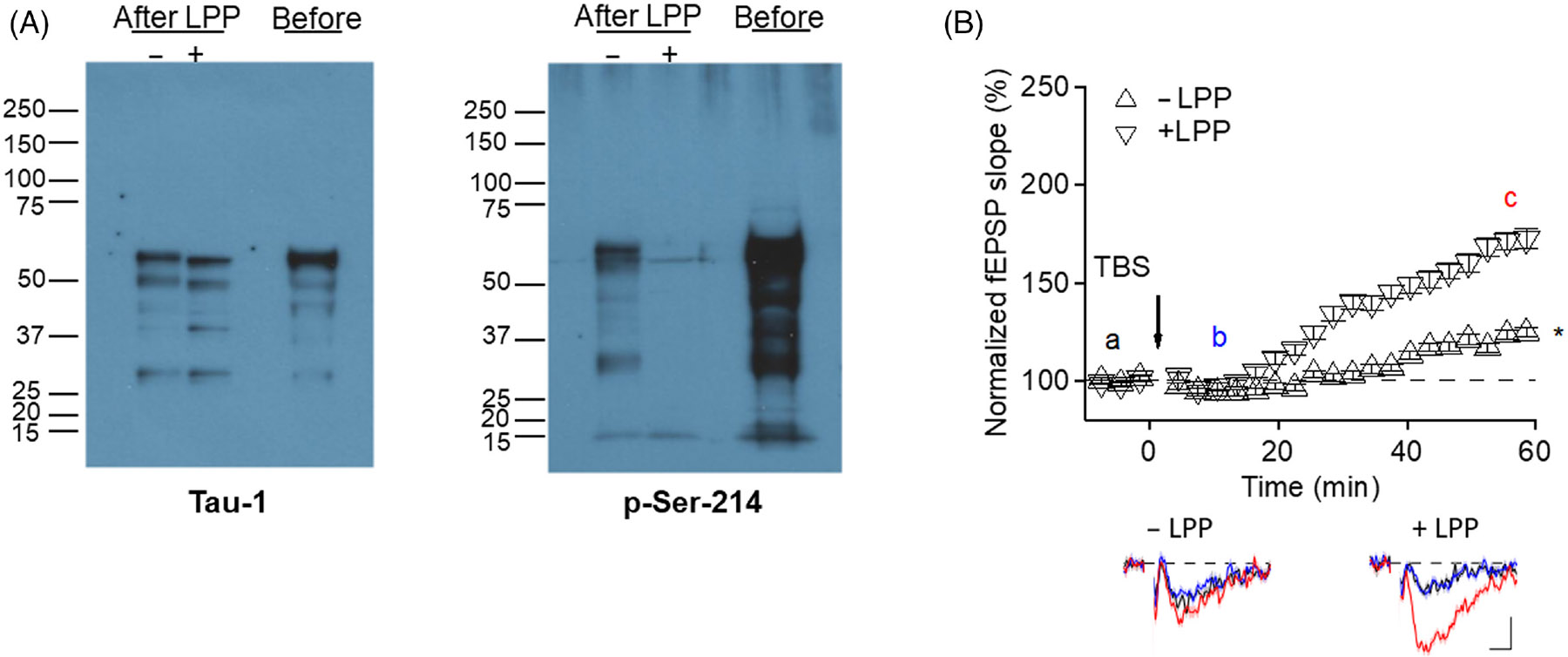
Phosphorylation of tau is required for inhibition of LTP. (A) PMVECs expressing the 1N4R form of endothelial tau were infected with ExoY^+^ bacteria, and supernatants were collected 6 h later. The untreated supernatant then was directly analyzed by immunoblot (Before) or was pretreated (After) with Lambda protein phosphatase (LPP; “+” = LPP treated sample, “−“= sample treated with reaction buffer alone) prior to being analyzed by immunoblot using either pan-tau antibody (Tau1) or Ser-214-tau antibody. LPP treatment abolished Ser-214 immunoreactivity. Molecular weights are in kDa. (B) Hippocampal LTP was abolished by supernatants collected following endothelial cell infection with ExoY^+^ bacteria. However, LPP treatment of the supernatant inactivated cytotoxicity of tau and rescued LTP to normal levels. The bottom shows representative averages of five traces obtained from timepoints “a” (pre-TBS; black), “b” (5–8 min post-TBS; blue), and “c” (57–60 min post-TBS; red). X-axis bar represents 2 ms and Y-axis bar represents 0.1 mV. * Denotes *p* ≤ .05 using ANOVA.

To assess whether phosphorylation plays any role in LTP suppression by endothelial-derived tau, supernatant collected from ExoY^+^-infected 1N4R expressing endothelial cells was treated with the broad-spectrum phosphatase from lambda phage (lambda protein phosphatase). As shown in Figure 7, lambda phosphatase treatment effectively dephosphorylated endothelial-derived tau. When phosphatase-treated supernatant was added to brain slices, LTP inhibition was abolished (Figure 7) demonstrating that tau phosphorylation, and perhaps phosphorylation of additional proteins released following infection, is required for inhibition of LTP.

**FIGURE 8:**
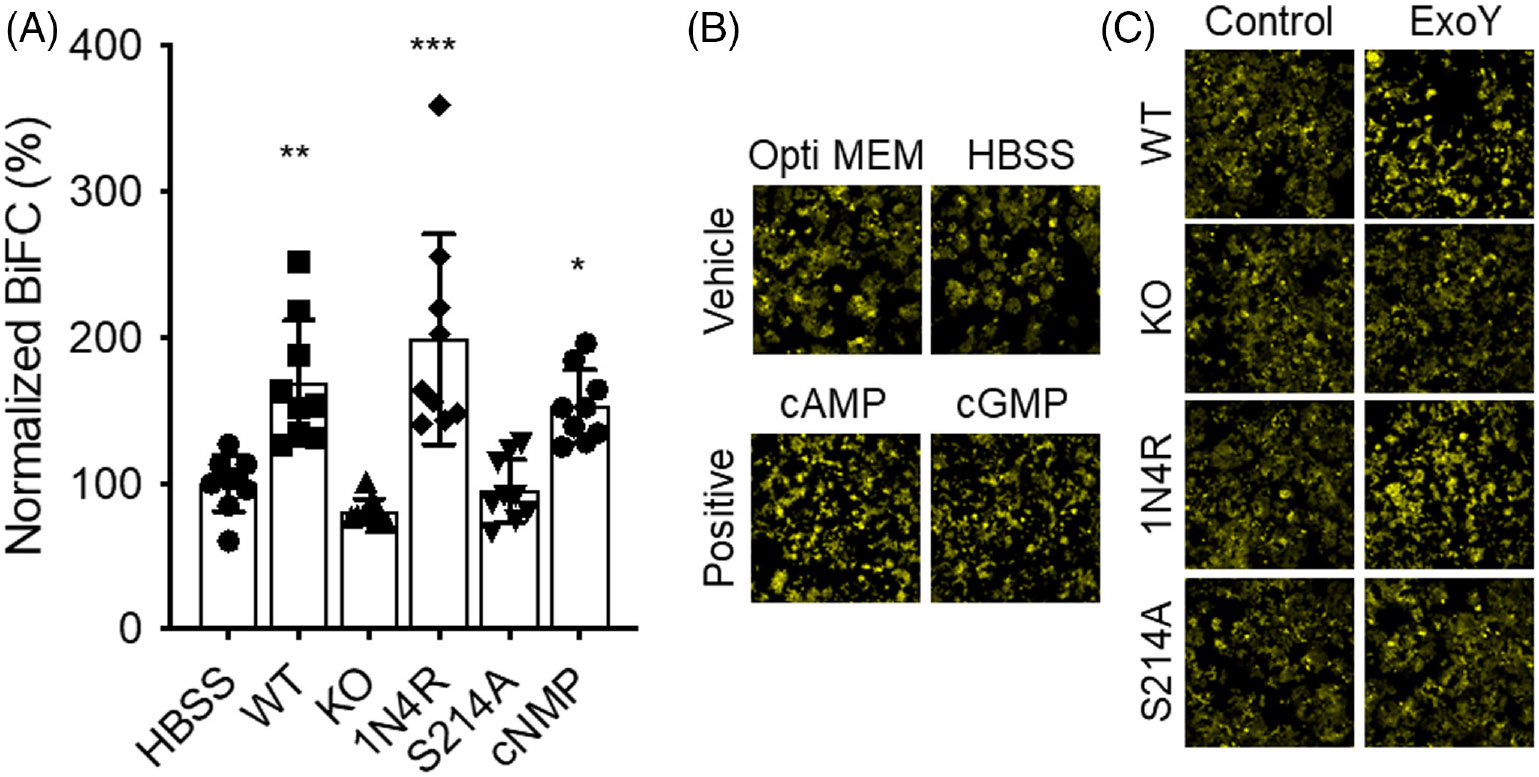
Ser-214 phosphorylation is required for endothelial tau-induced seeding of neuronal tau. (A) Quantitation of BiFC fluorescence following treatment of HEK293 cells with either HBSS (negative control; fluorescence level of these cells was set to 100%), membrane-permeant cyclic nucleotides (cNMP; positive control), or supernatant collected from wild-type (WT), tau knockout (KO), 1N4R tau-expressing (1N4R), or Ser-214 mutant (S214A)-expressing cells following ExoY^+^ infection (HBSS vs. WT **p ≤ .0043; HBSS vs. KO ns 0.8823; HBSS vs. 1N4R ****p ≤ .0001; HBSS vs. 1N4R-mutant ns 0.9997; HBSS vs. cNMP * 0.0494; *N* = 9). (B) Representative BiFC images of negative (Opti-MEM and HBSS) and positive (cAMP and cGMP) control cultures of HEK293 cells. (C) Representative BiFC images of HEK293 cells treated with supernatant collected from either untreated (Control) or ExoY^+^-infected (ExoY) wild-type (WT), tau knockout (KO), 1N4R, or Ser-214 mutant (S214A) tau-expressing cells.

A final set of studies was performed to assess whether Ser-214 phosphorylation is necessary to induce oligomerization of tau in cells. For these studies, HEK cells expressing BiFC-tagged fluorescent portions of the tau protein were treated with cell supernatants, and oligomerization of tau was quantified in treated cells 24 h later by increased fluorescence. As shown in Figure 8, treatment of cells with supernatant collected from either wild-type PMVECs or PMVECs expressing the 1N4R form of endothelial tau-induced tau oligomerization. Likewise, in cells where cyclic nucleotide levels were artificially increased by addition of membrane-permeant cyclic nucleotides, a slight but statistically significant increase in tau oligomerization was also detected. In contrast, tau oligomerization was not induced by supernatants collected from KO cells (Figure 8). Mutation of Ser-214 also abolished the capacity of secreted tau to induce tau oligomerization (Figure 8).

## 4 ∣ DISCUSSION

The role of Ser-214 phosphorylation of tau in the production of cytotoxic tau following infection of pulmonary endothelial cells by *Pseudomonas aeruginosa* was investigated. Key findings were that Ser-214 phospho-tau is responsible, in part, for pulmonary endothelial cell-derived toxicity. This was demonstrated in an in vitro cytotoxicity assay and in isolated perfused lungs. In contrast, Ser-214 phosphorylation of tau was not, yet global dephosphorylation of tau was, critical for the disruption of LTP. This result indicates that other forms of phosphorylated tau may be responsible for cognitive deficits that occur following pneumonia. Besides Ser-214, tau has multiple additional PKA consensus sites,^27,28^ as well as non-PKA phosphorylation sites,^29^ that may be critical for generation of neurotoxic forms of tau during pneumonia. Additional studies investigating the significance of tau isoforms and alternative phosphorylation sites of tau in the inhibition of LTP need to be performed.

Previously, we had used a construct encoding human brain tau to investigate the role of Ser-214 phosphorylation in the regulation of microtubule dynamics in PMVECs^12^ and demonstrated that Ser-214 phosphorylation of tau impacts microtubule assembly processes. Although tau is highly conserved across most of the protein, critical differences exist between human and rat tau, particularly in the N-terminal region of the protein.^21^ Previously, it had been demonstrated that the N-terminal region of tau is critical for the early events associated with tau misfolding during neurodegenerative processes.^30^ As such, constructs encoding rodent tau were used exclusively for these studies assessing the role of Ser-214 phosphorylation in the generation of toxic tau following infection of primary cultured rat PMVECs.

The results demonstrate that Ser-214 containing hyperphosphorylated forms of tau are cytotoxic to pulmonary endothelial cells (Figures 1 and 2), and that the production of Ser-214 containing forms of hyperphosphorylated tau are due to activation of PKA following infection by *P. aeruginosa* (Figure 3). Previously, PKA was identified as a potential modulator of endothelial tau phosphorylation, and it was determined that PKA phosphorylation of endothelial tau led to microtubule reorganization and the induction of endothelial gaps.^12,16^ These studies extend those observations by demonstrating that *P. aeruginosa* infection leads to PKA activation, which contributes to the generation of endothelial-derived cytotoxic forms of tau. The mechanisms leading to PKA activation following infection are incompletely understood, but the nucleotidyl cyclase activity of ExoY generates purine and pyrimidine cyclic nucleotides that are sufficient to induce PKA activation.^16^ However, the U and T exoenzymes transferred to target cells by the PA103 strain do not possess cyclic nucleotide synthetic activity, and instead possess phospholipase and ribosyl transferase activity, respectively. As such, the process leading to PKA activation during PA103 infection cannot be defined. One possibility that may lead to the activation of PKA by PA103 is ExoU cleavage of a membrane lipid could lead to the generation of a secondary messenger molecule that ultimately leads to PKA activation.^31-33^ This possibility deserves further investigation.

The results demonstrate that activation of PKA by membrane-associated adenylyl cyclases together with global inhibition of phosphodiesterases does not lead to the generation and secretion of toxic tau (Figure 4). This suggests that either the compartment in which cyclic nucleotides are generated or additional effects of bacteria following infection impact PKA targeting to tau. It is possible that the exoenzymes transferred during infection target tau directly, although there is no data to support this action. However, in the case of *P. aeruginosa* strain ExoY^+^, it is established that the ExoY exoenzyme is a promiscuous cyclase that also generates cGMP, cUMP, and cCMP, in addition to cAMP following infection.^15,16^ As such, it is likely that additional secondary messenger systems are activated following infection resulting in the generation of cytotoxic forms of oligomeric tau. In fact, it has been demonstrated that protein kinase G (PKG), which is activated by cGMP, can phosphorylate tau on Ser-214.^34^ Whether the additional cyclic nucleotides generated by ExoY can activate enzymes that phosphorylate tau has not been investigated.

The data demonstrate that Ser-214 tau directly disrupts the pulmonary endothelial barrier (Figure 5). This effect likely contributes to the pulmonary edema associated with pneumonia, and potentially may delay repair of the alveolar–capillary membrane following pneumonia. It is well-established that pneumonia during early childhood leads to pulmonary dysfunction that can last into adulthood (reviewed in Griimwood and Chang^35^), and more recent data from analysis of COVID-19 patients have determined that those patients experience severe long-term pulmonary deficiencies following their pneumonia including scarring, long-term bronchitis, dyspnea, and pulmonary vascular abnormalities that included vascular dilation and thrombosis.^36-40^ Whether tau contributes to any of these long-term morbidities has not been considered.

Previously, we had demonstrated that tau is essential for the disruption of LTP observed following pulmonary infection.^10^ The results presented here support that conclusion but indicate that Ser-214 phosphorylation is not essential in the early events leading to suppression of LTP (Figures 6 and 7). A potential explanation for these results is that it has been previously demonstrated that phosphorylation of tau at Ser-214 is protective for neurons and inhibits paired helical filament assembly and toxicity.^41^ As such, removal of Ser-214 forms of oligomeric tau might not be expected to impact LTP inhibition by lung endothelial-derived forms of cytotoxic tau. In addition, treatments that stimulate PKA signaling have been shown to stabilize LTP and memory perhaps, in part, due to Ser-214 phosphorylation of tau.^42-44^ The results reported here need to be interpreted conservatively, however, for multiple reasons, as it is possible that Ser-214 containing tau oligomers may have effects on the nervous system that would not be detected in LTP studies using acute brain slices. For example, endothelial-derived Ser-214 containing tau oligomers may injure endothelial cells in the brain, which might lead to coagulopathies leading to hypoxic brain injury,^45^ which has been shown to lead to elevated phospho-tau in brains.^46^ It is also notable that the Ser-214 mutant tau that was generated following endothelial infection was able to seed neuronal tau, providing an alternative explanation for why the mutant may have reduced LTP. In this case, it may be that the Ser-214 mutant form seeded dysfunctional neuronal tau, a possibility that needs to be explored. In addition to the outlined possibilities, we had previously demonstrated that tau oligomers cause long-term effects on neurons. Specifically, we have shown that 3–4 weeks postinfection the dendritic spine density is decreased in association with impaired learning and memory.^8^ Recordings using acute brain slices would not detect long-term effects on brain architecture. Finally, we focused our studies on the 1N4R form of endothelial tau, and it is conceivable that Ser-214 phosphorylation of the other tau isoforms may have effects on LTP. As such, additional studies investigating the effects of other forms of tau on LTP are warranted, and studies in progress are investigating these additional possibilities.

In summary, studies were conducted to assess the role of Ser-214 phosphorylation of tau in the generation of hyperphosphorylated tau that has been implicated as an agent responsible for the long-term consequences associated with pneumonia caused by *P. aeruginosa*. The results demonstrate that Ser-214 tau causes endothelial cell death and pulmonary endothelial barrier breakdown. In contrast, Ser-214 phosphorylation of the 1N4R tau isoform is not sufficient to inhibit hippocampal LTP. The demonstration that multiple subtypes of tau are liberated during pneumonia, and that these different subtypes may be responsible for unique clinical symptoms observed because of pneumonia, highlights the complexity of developing clinical treatments for addressing the long-term deleterious effects of pneumonia.

## Data Availability

No datasets were generated during the current study, so data sharing is not applicable.

## Abbreviations:

aCSF: artificial cerebrospinal fluid
ANOVA: analysis of variance
BiFC: bimolecular fluorescence complementation measurement
DMEM: Dulbecco's Modified Eagle's Medium
Exo: exoenzyme
fEPSP: field excitatory postsynaptic potential
HBSS: Hank's Balanced Salts Solution
$K_{f}$: filtration coefficient
KO: knockout
LPP: lambda protein phosphatase
LTP: long-term potentiation
PKA: protein kinase A
PKG: protein kinase G
PKI: protein kinase A inhibitor peptide
PMVECs: pulmonary microvascular endothelial cells
